# HMGA2 overexpression predicts relapse susceptibility of blastemal Wilms tumor patients

**DOI:** 10.18632/oncotarget.23256

**Published:** 2017-12-14

**Authors:** Lourdes Hontecillas-Prieto, Daniel J. García-Domínguez, Rosa García-Mejías, Gema L. Ramírez-Villar, Carmen Sáez, Enrique de Álava

**Affiliations:** ^1^ Institute of Biomedicine of Seville (IBiS), Pathology Unit, Hospital Universitario Virgen del Rocío/CSIC/Universidad de Sevilla, CIBERONC, Seville, Spain; ^2^ Pediatric Oncology Unit, Hospital Universitario Virgen del Rocío/CSIC/Universidad de Sevilla, Seville, Spain

**Keywords:** Wilms tumors, embryonic stem cell markers, HMGA2, blastemal stratification

## Abstract

Wilms tumor (WT) is an embryonal malignant neoplasm of the kidney that accounts for 6–7% of all childhood cancers. WT seems to derive from multipotent embryonic renal stem cells that have failed to differentiate properly. Since mechanisms underlying WT tumorigenesis remain largely unknown, the aim of this study was to explore the expression of embryonic stem cell (ESC) markers in samples of WT patients after chemotherapy treatment SIOP protocol, as the gene expression patterns of ESC are like those of most cancer cells. We found that expression of ESC markers is heterogeneous, and depends on histological WT components. Interestingly, among ESC markers, HMGA2 was expressed significantly stronger in the blastemal component than in the stromal and the normal kidney. Moreover, two subsets of patients of WT blastemal type were identified, depending on the expression levels of HMGA2. High HMGA2 expression levels were significantly associated with a higher proliferation rate (p=0.0345) and worse patient prognosis (p=0.0289). The expression of HMGA2 was a stage-independent factor of clinical outcome in blastemal WT patients. Our multivariate analyses demonstrated the association between LIN28B–LET7A–HMGA2 expression, and the positive correlation between HMGA2 and SLUG expression (p=0.0358) in blastemal WT components. In addition, patients with a poor prognosis and high HMGA2 expression presented high levels of MDR3 (multidrug resistance transporter). Our findings suggest that HMGA2 plays a prominent role in the pathogenesis of a subset of blastemal WT, strongly associated with relapse and resistance to chemotherapy.

## INTRODUCTION

Wilms tumor (WT) or nephroblastoma is the most common pediatric renal tumor and represents 6% of all childhood cancers. About 75% of WT are diagnosed before the age of 5 years [1]. Treatment with surgery, chemotherapy, and/or radiotherapy has greatly improved the prognosis of children with WT and the 5-year disease-free survival rate has increased from 30% to 85% [2–6]. However, these therapies are associated with significant short- and long-term morbidity and there remain a substantial proportion of children who relapse [7–10].

Two groups have made major contributions to the management of WT, the National Wilms Tumor Study Group (NWTS) and updated by the Children’s Oncology Group (COG), and the International Society of Pediatric Oncology (SIOP). Each developed a different protocol for the diagnosis and treatment of WT [11–13]. The COG in North America developed a protocol based on upfront surgery (nephrectomy) to accurately assess the tumor stage and histology [14–15]. In Europe, SIOP delays nephrectomy for 4–6 weeks, favouring upfront chemotherapy to minimize complications of surgery and tumor spillage [14, 16]. Although different, both treatment approaches have shown almost equivalent clinical outcomes [15, 17–18].

WT is an embryonal tumor that is thought to arise from aberrant differentiation of multipotent renal progenitors that are fated to become the kidney and escape pathways of normal epithelial conversion [19]. WT emerges within precursor lesions known as nephrogenic rests that resemble embryonic developmental stages not found in the postnatal kidney [20]. An expression analysis study with a wide set of WTs identified five subsets of WT with different clinical and pathologic features that showed evidence of disruption at different times during normal development [21]. This evidence suggests that the block in the nephrogenic process is an early, maybe even initiating, step in the formation of WT. Thus, several studies have shown an overlap between WT and embryonic kidney gene expression profiles [22–24], linking Wilms tumorigenesis to defective embryonic development [25–26]. In fact, the blastemal component has been described as resembling the metanephric mesenchyme of the human fetal kidney, where embryonic renal stem cells reside [27].

It has been described that the comparison of gene expression patterns between embryonic stem cells (ESC) and cancer cells reveals shared features [28]; and different types of tumors have shown an increase in ESC markers (such as HMGA2 (High-mobility group AT-hook 2), KLF4 (Kruppel Like Factor 4), NOTCH1) that play a role in pluripotency, self-renewal and tumorigenic effects [29–32]. *Hmga2* is expressed in a variety of malignant tumors [33–34] and in undifferentiated cells during embryogenesis [29]. The overexpression of HMGA2 is correlated with the down-regulation of let-7 miRNAs and the overexpression of LIN28B [35–36]. The tumor-suppressive role of the let-7 miRNA family is antagonized by the self-promoting oncogenic triangle composed of the transcription factor HMGA2, the Insulin-like growth factor-2 mRNA-binding proteins (IGF2BP1) and LIN28B [37]. The LIN28-Let-7-HMGA2-IGF2BP pathway has been broadly implicated in the regulation of stem cell function and is active in a broad range of cancers [38–39] induces aberrant proliferation, self-renewal and 2D/3D migration [37].

Given the similarity between cancer cells and ESC on the one hand and the link between kidney development and Wilms tumorigenesis on the other hand, we proposed that ESC markers play a role in tumor aggressiveness and that there are pathways that remain active after neoadjuvant therapy. To our knowledge, this study represents the first report of Let7 targets of the oncogenic triangle of Lin28B-HMGA2-IGF2BP1 in WT, and the strong association of HMGA2 with relapse and resistance to chemotherapy. It suggests that this pathway is an emerging and potential therapeutic target in WT treatment.

## RESULTS

### Differential expression of embryonic stem cell markers between normal kidneys and Wilms tumor samples

**Figure 1:**
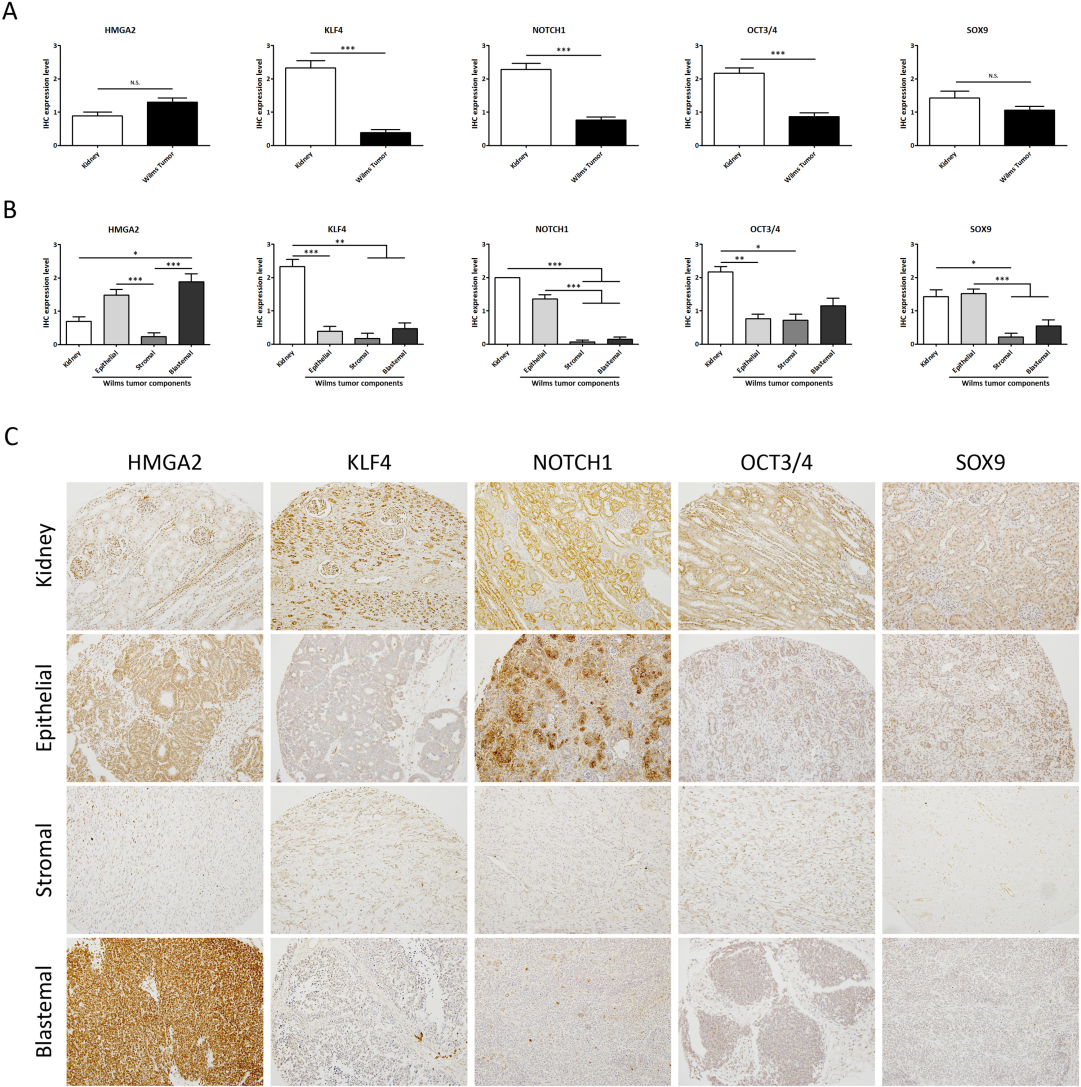
Analysis of the expression levels of embryonic stem cell markers showed differences between normal kidneys and WT samples **(A)** Expression analyses of 13 kidneys versus 70 WT samples (regardless of subcomponents conformation) by tissue microarray. **(B)** Differential embryonic stem cell expression in the epithelial, stromal, and blastemal WT components. **(C)** Representative immunohistochemical detection of embryonic stem cell markers in different WT components from tissue microarray (20X magnification). For all ANOVA analyses, ^*^ p<0.05; ^**^ p<0.01; ^***^ p<0.001. NS, not significant.

To characterize ESC marker expression in WT, we compared it between kidneys and tumors. A total of 83 formalin-fixed paraffin-embedded (FFPE) samples in the tissue microarray (TMAs) (Series 2) were analysed using immunohistochemical detection with antibodies against the selected ESC markers: High-mobility group AT-hook 2 (HMGA2), Kruppel Like Factor 4 (KLF4), NOTCH1, Octamer-binding Transcription factor 3/4 (OCT3/4) and SRY-Box 9 (SOX9). Those ESC markers are known to play a key role in pluripotency, self-renewal [29–32], kidney development and tumorigenesis by maintaining stem cell-like features [40–42]. We found that expression of KLF4, NOTCH1, and OCT3/4 was significantly lower in WT samples than in their normal kidney counterparts. However, we did not find any significant differences in HMGA2 and SOX9 expression between normal kidney and WT (Figure 1A).

Then we proceeded to evaluate differential expression of ESC markers in the components (epithelial, stromal, and blastemal) of WT samples. We observed a differential expression pattern in each tumor component. KLF4 and OCT4 had a very low expression in all the tumor components with respect to the kidney; in contrast, there were expression differences between components for the other three markers. The expression of HMGA2 was significantly higher in the blastemal components with respect to the other WT components and the kidneys. NOTCH1 and SOX9 showed a similar expression level in the epithelial WT component with respect to the kidneys, whereas there was no expression in the stromal and blastemal WT components (Figure 1B and 1C). Of note, NOTCH1 and SOX9 were expressed in the epithelial WT component with a significant correlation (p=0.0061) between them (data not shown). Many studies showed the relationship and role of a Notch1-Sox9 signaling axis [43–44] in different tumors. These TMA results indicate the relevance of ESC expression in the WT components.

### HMGA2 expression can be used to stratify blastemal Wilms tumor patients

A previous study suggested that two subsets of patients with blastemal WT exist, each one presenting a different sensitivity to chemotherapy [45]. To confirm if there were two subsets of blastemal WT with regards to expression of HMGA2, we studied its expression by Reverse transcription polymerase chain reaction (qRT-PCR) and immunohistochemistry (IHC) in two independent series of blastemal WT patients.

**Figure 2:**
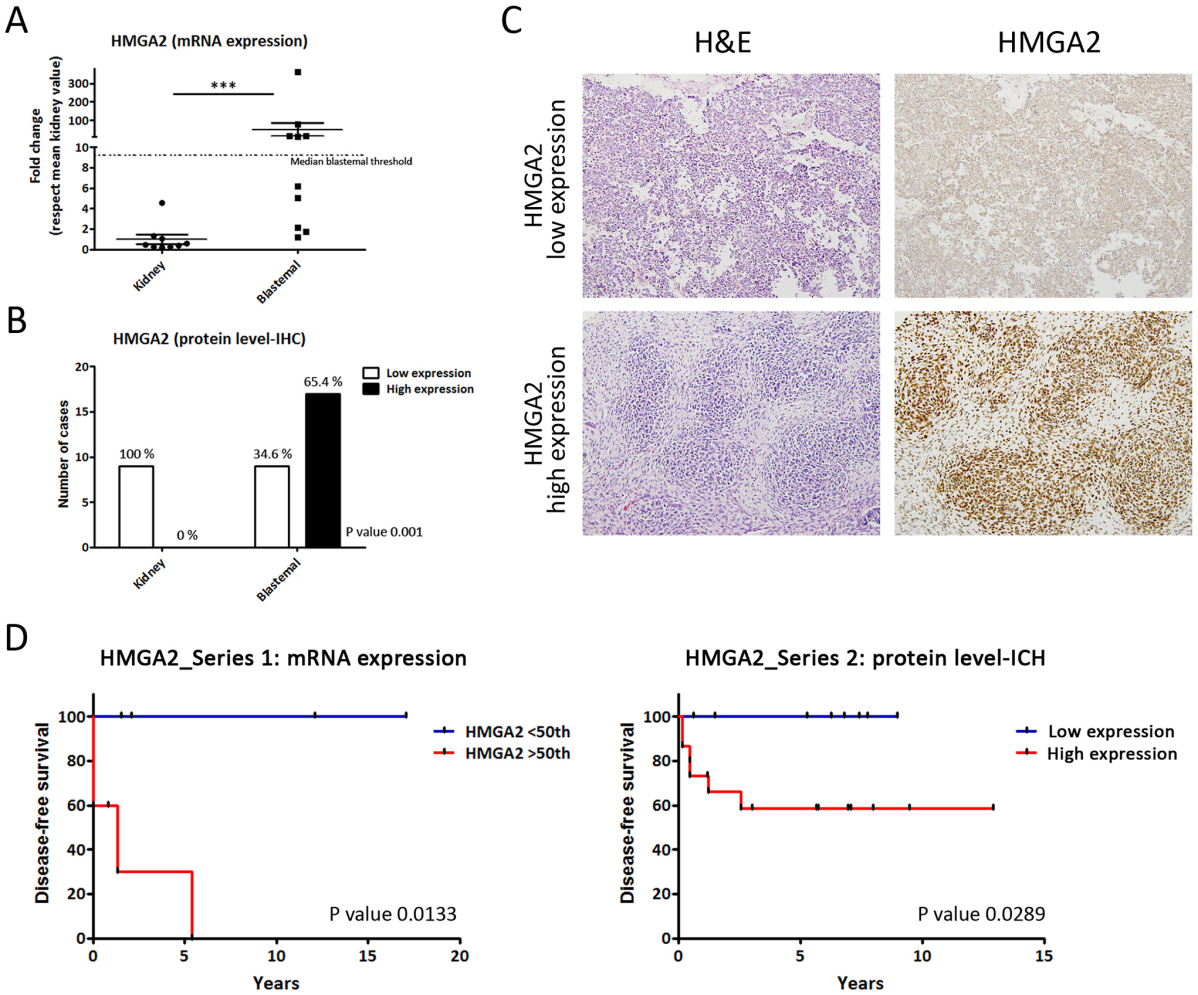
Differential HMGA2 expression distinguishes two types of blastemal WT component **(A)** Significant differences between the expression of *HMGA2* in kidneys and two blastemal WT components by qRT-PCR. The median cut-off value in blastemal WT samples was 9.2 (fold change). **(B)** Differential *HMGA2* expression between kidneys and two blastemal WT components by immunohistochemistry. **(C)** HMGA2 immunohistochemical detection in blastemal WT patient samples from tissue microarray (20X magnification). **(D)** HMGA2 Kaplan–Meier plot with disease-free survival according to the transcript and IHC expression in blastemal WT components showed statistically significant differences between low and high HMGA2-expressing tumors. For all the analyses, ^*^ p<0.05; ^**^ p<0.01; ^***^ p<0.001. NS, not significant.

First, we performed gene expression analysis of *HMGA2* in 11 WT and 11 kidneys frozen samples (Series 1). Then, the levels of mRNAs encoding *HMGA2* in WT were compared with their kidney-paired samples by qRT-PCR. The dot graph shows (Figure 2A) that *HMGA2* mRNA levels were significantly higher in blastemal WT than kidneys (p=0.0003). Applying the median HMGA2 cut-off in blastemal WT included in Series 1, we identified a blastemal WT group with high *HMGA2* expression (equal or greater than a 9-fold change) and a group with low *HMGA2* expression (similar to kidney) (Figure 2A). Next, we analysed HMGA2 expression in Series 2 by IHC in blastemal WT samples (Figure 2B and 2C). Similar to the analysis of mRNA levels, we observed a differential expression between kidneys and blastemal WT, in which 100% of kidney samples showed low HMGA2 expression, whereas the blastemal WT component was divided into two groups showing statistically significant differences in HMGA2 expression (p=0.001): 65.4% of blastemal WT samples had high levels of HMGA2 compared with 34.6% with low expression (Figure 2B and 2C). Therefore, HMGA2 expression could be useful for distinguishing two subgroups of blastemal WT.

The last step was to correlate these two subsets of blastemal WT patients with prognosis. In Series 1, we analysed the relationship between disease-free survival and HMGA2 transcript expression levels by Kaplan–Meier test. We used a median cut-off because it is more representative and less biased. We observed that patients with high *HMGA2* transcript expression showed a higher relapse rate than patients with low *HMGA2* expression in the blastemal WT component (p=0.0133) (Figure 2D). An analogous result was obtained in Series 2 studying protein expression levels by IHC.

Our results showed that WT patients with high expression of *HMGA2* had a significantly worse prognosis than WT patients with low mRNA levels (p=0.0289). In fact, the patients in the subgroup with low expression did not relapse in any series (Figure 2D). The results obtained in the epithelial WT component failed to show any impact of *HMGA2* expression in clinical outcome (Supplementary Figure 1A). In conclusion, HMGA2 overexpression could be an important prognostic biomarker in blastemal WT patients associated with shortened recurrence-free survival.

### HMGA2 expression was independent of tumor stage and SIOP-treatment

**Figure 3:**
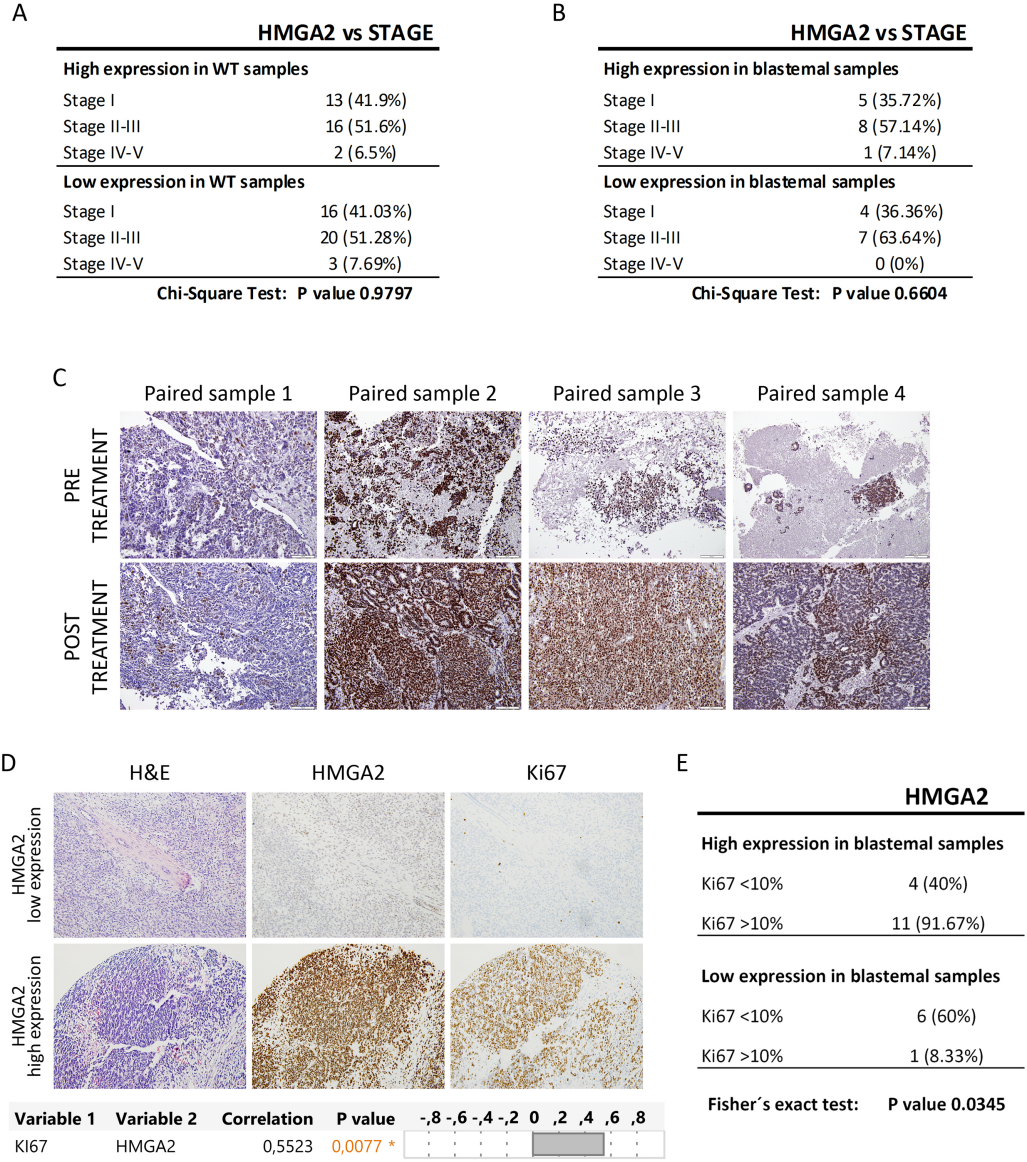
The expression of HMGA2 did not correlate with tumor stage and SIOP-treatment protocol, but correlated with Ki-67 expression **(A)** Relation between HMGA2 expression and stage in all WT subtypes. **(B)** No significant relationship between high and low expression of HMGA2 and stage in blastemal WT component. **(C)** Similar HMGA2 expression between pre- and post-treatment paired WT samples by immunohistochemistry. **(D)** Immunohistochemical detection and significant positive correlation between HMGA2 and Ki-67 in blastemal WT components (20X magnification) (upper panel). Correlation analyses between HMGA2 and Ki-67 (lower panel). **(E)** Significant relationship between high and low expression of HMGA2 and Ki-67 in blastemal WT component.

Stage is a classical prognostic factor for WT patients [46], we then evaluated whether HMGA2 expression was related with tumor stage. On the one hand, we confirmed that stage correlated with worse prognosis in our patient Series 2 by Kaplan–Meier test (Supplementary Figure 2A). One the other hand, we analysed the relation between HMGA2 expression and tumor stage, and we observed that HMGA2 expression did not correlate with stage in all WT subtype (p=0.9797) and in blastemal WT component (p=0.6604) (Figure 3A and 3B). So, HMGA2 was a tumor stage independent prognostic factor associated with high incidence of tumor recurrence in blastemal WT patients.

Then, we wanted to determine whether HMGA2 expression could be modified by treatment, since our study was based on SIOP protocol. We were able to found in our Biobank four paired samples before and after treatment. One paired sample was the pre- and post-treatment tumor, and the other three paired samples were fine-needle aspiration cytopathology samples taken prior to neoadjuvant chemotherapy and their post-treated tumors. We observed: i) low HMGA2 expression in paired pre- and post-treatment sample 1; ii) high HMGA2 expression mainly in the blastemal component in both type of paired samples 2 and 3, and iii) moderate expression in epithelial and blastemal component in paired sample 4 (Figure 3C). These results would suggest that the expression of HMGA2 does not seem to depend on the specific treatment protocol (SIOP vs COG) for WT.

Finally, we evaluated Ki-67 expression as a proliferative marker in WT [47] in Series 2. Ki-67 immuno-stain was semi-quantitatively scored as <10% or >10% for nuclear expression in WT components. We obtained a significantly positive correlation (p=0.0077) between Ki-67 and HMGA2 expression in the blastemal WT component (Figure 3D). Fisher’s exact test confirmed a statistically significant difference in proliferation in high-HMGA2 expressing blastemal tumors – 91.67% of high-HMGA2 blastemal tumors were more proliferative (>10% Ki-67 expression) compared with only 8.33% of low-HMGA2 blastemal tumors (Figure 3E). Nevertheless, HMGA2 expression in the epithelial component did not correlate with proliferation rate (Supplementary Figure 1B). So, only blastemal WT samples with high HMGA2 expression were more proliferative.

In conclusion, HMGA2 overexpression was not correlated with stage and SIOP treatment protocol, and its high expression was associated with a higher proliferation rate in blastemal WT.

### *HMGA2* significantly correlates with *LIN28B–LET7A* and SLUG expression

It is known that *HMGA2* overexpression is correlated with the down-regulation of *let-7* miRNAs [36] and with overexpression of LIN28B [35] in several tumor types. HMGA2 has also been implicated in the regulation of the expression of transcription factors such as Snail, Slug, ZEB1, and ZEB2 [48]. Urbach et al., described that *LIN28B* overexpression sustains early renal progenitors and promotes WT formation [49], thus we analysed the gene expression of *LIN28 (A, B), LET7 (A, B, C), HMGA2*, and *SLUG* genes in frozen blastemal WT samples (Series 1).

**Figure 4:**
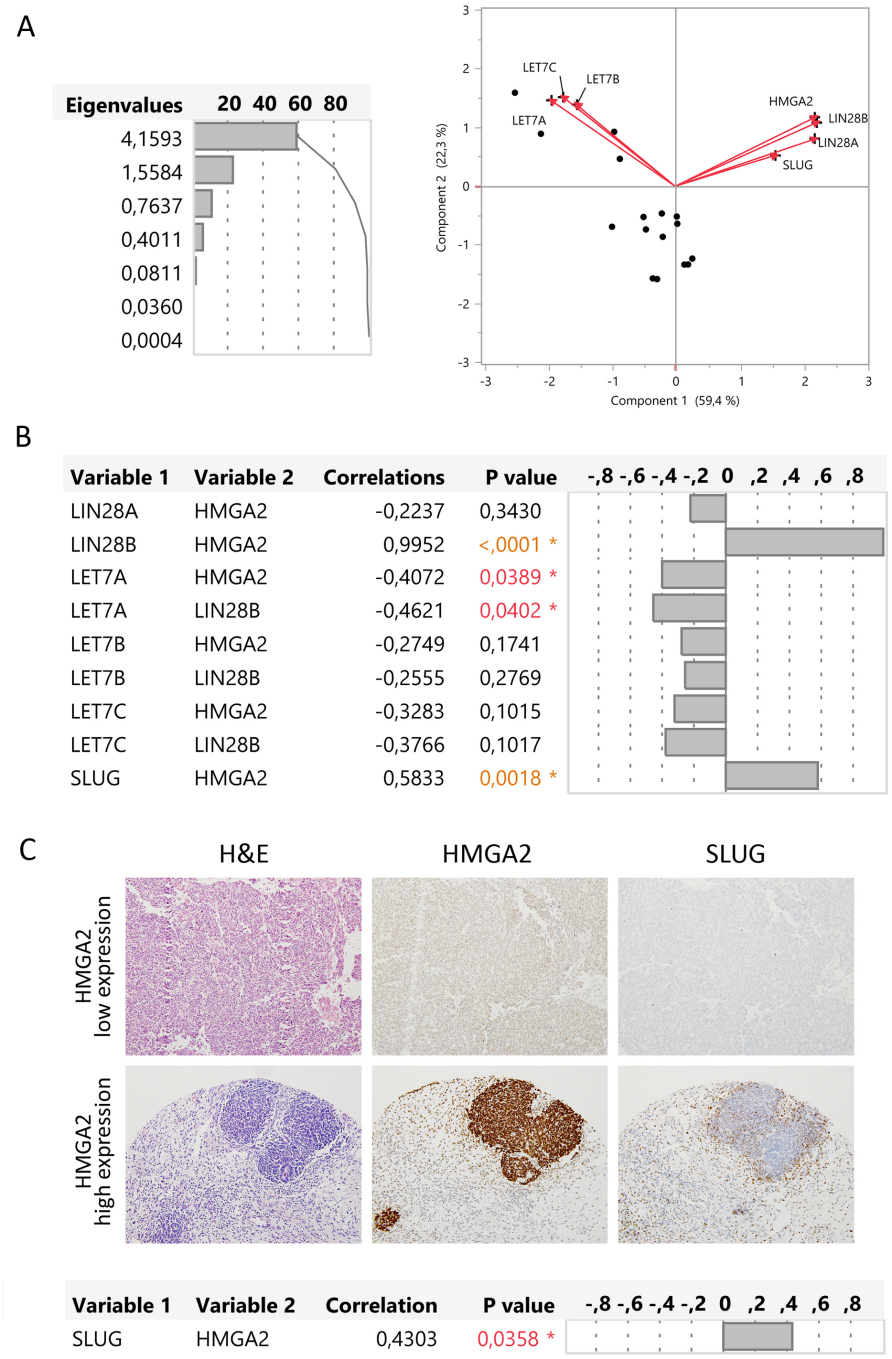
*HMGA2* correlated with *LIN28B, LET-7A*, and *SLUG* gene expression in the blastemal WT component **(A)** Principal component analysis represented by biplot showed the possible relations between genes. **(B)** Multivariate correlations between the expression of *HMGA2, LIN28A, LIN28B, LET7A, LET7B, LET7C*, and *SLUG* genes. **(C)** Immunohistochemical correlation between HMGA2 and SLUG in two blastemal WT components from tissue microarray (20X magnification).

We performed a multivariate analysis and the principal component analysis showed that *HMGA2, LIN28A, LIN28B*, and *SLUG* had a positive relation and that these genes had a negative relation with *LET7A, B*, and *C*. To confirm these results, we analysed the correlation between gene pairs using the multivariate Spearman’s test. We observed a positive correlation between *LIN28B* and *HMGA2* (p<0.0001) but not between *LIN28A* and *HMGA2* (p=0.3430) (Figure 4A). So, *LIN28B* is differentially expressed in blastemal WT like *HMGA2*, but *LIN28A* does not appear to play a relevant role. On the other hand, only *LIN28B* significantly suppressed the expression of *LET7A* (p=0.0402) and only *LET7A* had a significant negative correlation with *HMGA2* (p=0.0389) (Figure 4B).

In addition, the multivariate analysis showed a significantly positive correlation between *HMGA2* and *SLUG* expression levels (p=0.0018). We evaluated SLUG expression in low and high HMGA2-expressing blastemal WT samples from Series 2 by IHC. High-HMGA2 blastemal tumors showed a high SLUG expression, whereas low-HMGA2 blastemal tumors showed low SLUG expression levels. We confirmed the positive correlation between HMGA2 and SLUG (p=0.0358) in the blastemal WT component (Figure 4C). Therefore, we defined two blastemal WT subgroups according to HMGA2 expression and identified a potential regulatory pathway *HMGA2*-*LIN28B–LET7A* as well as a correlation between HMGA2 and SLUG.

### The blastemal WT subgroup with HMGA2 and MDR3 overexpression shows a higher relapse rate

**Figure 5:**
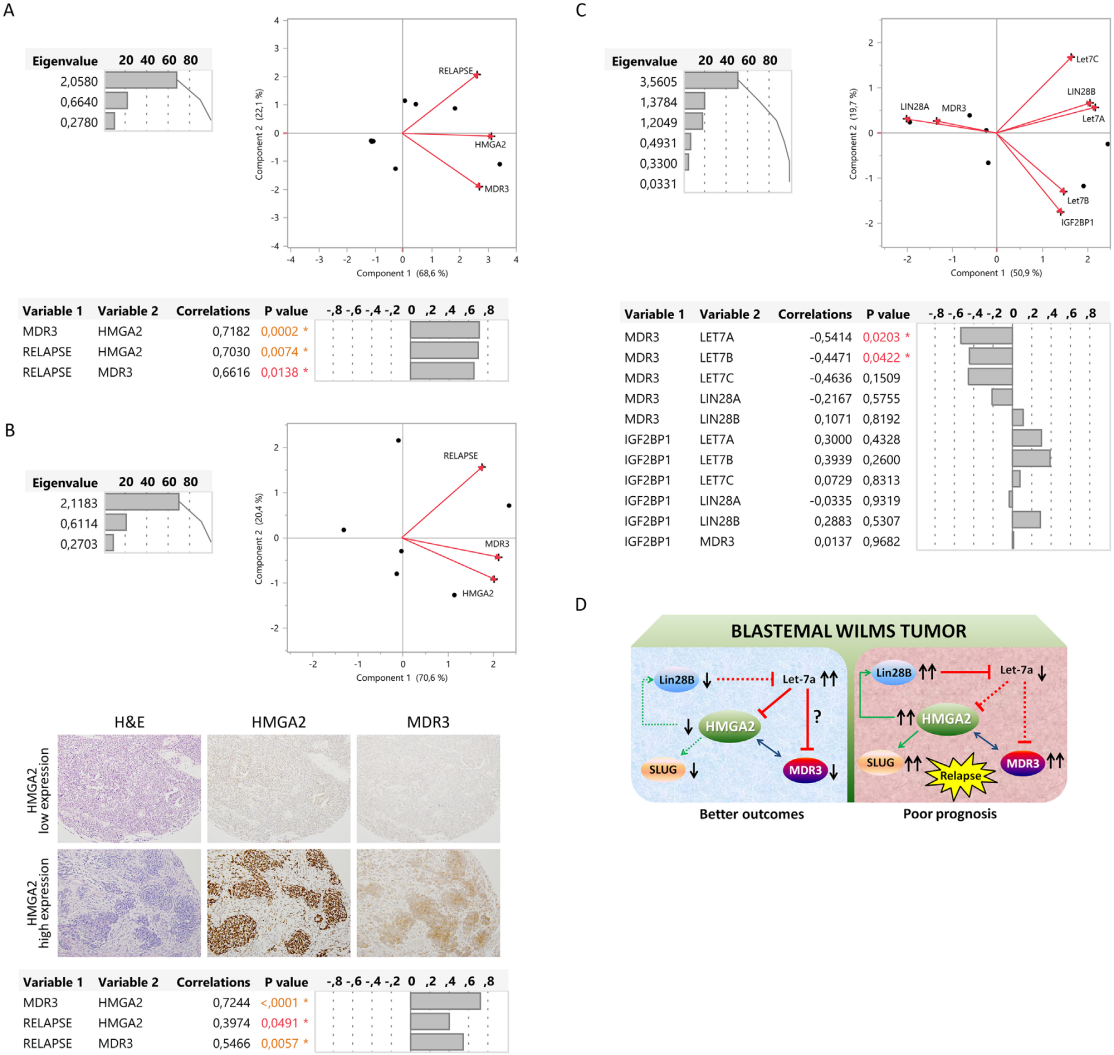
Description of relationship between LIN28, LET7, HMGA2, and MDR3 as a possible relapse regulatory pathway in blastemal WT **(A)** Significantly positive correlation by multivariate analysis (principal component and multivariate correlation) between *HMGA2*, *MDR3* and relapse in two blastemal WT patients according to mRNA tumor expression. **(B)** Significantly positive correlation by multivariate analysis (principal component and multivariate correlation) between *HMGA2, MDR3*, and relapse in two blastemal WT patients from tissue microarray (20X magnification). **(C)** Multivariate analysis between *MDR3* and *LIN28–LET7–IGF2BP1* in two blastemal WT patients according to the transcript tumor expression. **(D)** Representation of the HMGA2 alternatives pathways in different subgroups of blastemal WT with respect to prognosis.

We previously identified two types of blastemal WT dependent on MDR3 (multidrug resistance transporter) expression, and reported that blastemal WT patients with high MDR3 expression had a poor prognosis [45]. In this study, we evaluated if there was a relationship between HMGA2 and MDR3 in the blastemal WT component using two independent patient series. Based on expression levels (mRNA and protein), we performed a multivariate analysis with HMGA2, MDR3, and WT relapse. Principal component analysis showed a positive relationship between the three parameters, and multivariate Spearman’s test confirmed the positive correlation between MDR3-HMGA2 (p=0.0002 and p<0.0001), relapse-HMGA2 (p=0.0074 and p=0.0491), and relapse-MDR3 (p=0.0138 and p=0.0057), for RNA and protein, respectively (Figure 5A and 5B). Thus, we demonstrated in two independent series that patients with high expression of HMGA2 also expressed high levels of MDR3 and these patients relapsed.

Boyerinas et al., described in ovarian cancer cells that let-7 could be implicated in the regulation of drug resistance, mainly in patients with recurrent disease who had undergone chemotherapy. They identified MDR1 as an indirect target of let-7 through the down-regulation of IGF2BP1 [50]. To this end, we analysed if *MDR3* expression correlated with *LIN28, LET7*, and/or *IGF2BP1* genes. By multivariate analysis we observed that *MDR3* expression in the blastemal component had a significant negative correlation with *LET7A* (p=0.0203) and *LET7B* (p=0.0422); the other genes (*LIN28A* and *B* and *IGF2BP1*) did not show any relationship with *MDR3* (Figure 5C). Therefore, high *HMGA2* expression in the blastemal WT component correlated with high *MDR3* and relapse, and high *MDR3* expression correlated with low *LET7A* and *LET7B* expression.

## DISCUSSION

WT is classified as an embryonal tumor and is postulated to derive from multipotent embryonic renal stem cells that have failed to differentiate properly [51–52]. The differentiation failure results in a similar histologic appearance of WT components to the fetal kidney [22, 53] (blastemal, stromal, and epithelial components). Embryogenesis and tumorigenesis share multiple regulatory mechanisms in several tumors, including WT [22–24]. Accordingly, embryonic stem cells and cancer cells have been reported to share a common gene expression pattern [28], and the expression of ESC markers (such as HMGA2, KLF4, NOTCH1, OCT3/4 and SOX9) that are known to play a key role in pluripotency, self-renewal [29–32], and cancer, is increased in many tumors [54–55]. Our results show that only KLF4, NOTCH1, and OCT3/4 were down-regulated in the whole tumor compared with normal kidney samples. However, a differential ESC expression pattern was observed in different WT components. In fact, we observed overexpression of HMGA2 in the blastemal WT component.

We focused our attention on the high HMGA2 expression in the blastemal and epithelial components and found that tumors with high HMGA2 expression in the blastemal component were more proliferative and presented a poor outcome with a higher patient relapse rate. These results could be explained because the blastemal, but not the epithelial component resembles the metanephric mesenchyme of the human fetal kidney where embryonic renal stem cells arise [27], and only the blastemal component preserves the molecular events implicated in WT onset [56]. Hence, this study and previous publications highlight the relevant role of the blastemal component in WT [53, 56–60].

We have described two groups of blastemal WT patients according to HGMA2 expression. We observed that HMGA2 expression is a stage-independent predictive factor of clinical outcome in blastemal WT patients, and relapse is correlated with a higher HMGA2 expression in blastemal WT (confirmed in both independent patient series used in this study). Several studies on human cancers have indicated that HMGA2 overexpression predicted poor prognosis without a significant correlation with tumor stage [33–34]. In contrast, Maschietto et al. did not observe a differential expression of HMGA2 in a previous gene expression analysis of blastemal WT components [61]. This fact could be explained because they compared fetal kidney (a good control to identify conserved genes in the earliest stages of kidney embryogenesis) with WT in COG American protocol [61]. Thus, HMGA2 could be a useful marker for stratifying blastemal WT into two groups with different clinical outcomes. However, further studies are necessary to clarify if the specific treatment protocol (SIOP vs COG) for WT would modify or not the expression of HMGA2 and its prognostic value.

One key result from this study was the multivariate analysis between different components of the oncogenic triangle Lin28B-HMGA2-IGF2BP1 and Let7 in the WT blastemal component. HMGA2 overexpression is correlated with the down-regulation of let-7 miRNAs and the overexpression of LIN28B [35–36], and multivariate analyses showed that *LIN28B*, but not *LIN28A*, was negatively correlated with *LET7A*. Moreover, the down-modulation of microRNA *LET7A* up-regulated *HMGA2* expression in blastemal components in which *HMGA2* was overexpressed (the expression was reversed in blastemal components with low *HMGA2* expression). In accordance with our results, Urbach et al. revealed LIN28B upregulation in 19% of WT in two independent series of patients, compared with normal kidneys, but did not detect LIN28A expression in any sample. They suggested an association between LIN28B expression and relapse and death [49]. In our blastemal WT patient series, *LIN28B* was not involved in prognosis (Supplementary Figure 3A). As *HMGA2* overexpression associated with a poor outcome (more proliferation and relapse), but the upregulation of *LIN28B* was not involved in the outcome, our data suggest the existence of a feedback loop in which HMGA2 regulates the Lin28B-let7 pathway. In agreement with our hypothesis, several groups described a positive feedback loop in which HMGA2 further augments Lin28-let7-mediated gene expression *in vitro* [62] and others showed that HMGA2 regulated Lin28 genes [63]. Our results suggested that HMGA2 could have a predominant role in the oncogenic triangle, at least compared with LIN28B, due to its correlation with poor prognosis.

The major problems for WT patients with poor prognosis are relapse [7–10] and resistance to standard chemotherapy [58]. High MDR3 expression is associated with blastemal WT and poor prognosis, and to support this, we observed a significantly positive correlation between HMGA2-MDR3-relapse in two independent series of WT patients. In addition, HMGA2 has been described to induce SLUG expression [64], and in this study, we observed a positive correlation between HMGA2 and SLUG. In accordance with our results, both HMGA2 and SLUG overexpression have been reported to promote and contribute to cancer progression [64–67]. Moreover, human ESC express multidrug resistance transporters [68]. Therefore, high HMGA2 expression might promote disease progression through the up-regulation of SLUG, and HMGA2-MDR3 up-regulation could be related with resistance to chemotherapy. All these events may contribute to a poor prognosis in relapsed WT patients. In terms of the resistance to chemotherapy, other groups demonstrated a correlation between loss of let-7 and resistance to either chemotherapeutic drugs or radiation [64, 69–71]. Boyerinas et al., described *in vitro* that let-7 could be implicated in the regulation of MDR1-mediated resistance through the down-regulation of IGF2BP1 [50]. However, our multivariate analysis revealed that *IGF2BP1* was not implicated in the regulation of MDR3 in WT.

In summary, our findings suggest that HMGA2 plays a prominent role in the aggressiveness of blastemal WT component and could be a new biomarker for stratification. We propose a HMGA2 pathway in blastemal WT patients in which patients with high levels of HMGA2 exhibit poor prognosis (Figure 5D). In the model, HMGA2 induces LIN28B expression and consequently down-regulates LET7A, driving a regulatory loop. The down-regulation of LET7A and up-regulation of HMGA2 correlate with (and possibly induce) high MDR3 expression levels leading to an enhanced resistance to chemotherapy in those patients. Moreover, HMGA2 increases SLUG expression, promoting or contributing to tumor progression. It is interesting to note that the pro-oncogenic activity of the triangle is demonstrated in many types of tumors. Pathways that remain active after neoadjuvant therapy such as the oncogenic triangle Lin28B-HMGA2-IGF2BP1 and Let7, can be present in WT. Specifically, HMGA2 has a strong association with relapse and resistance to chemotherapy in blastemal WT, so it could be an emerging and potential therapeutic target in WT treatment.

## MATERIALS AND METHODS

### Patients and clinical samples

This retrospective study includes two independent series of samples with favorable histology (no foci of anaplasia), obtained between 1993 and 2016. Series 1 comprised 11 kidneys and 11 WT blastemal type frozen samples (paired samples). Series 2 comprised a tissue microarray (TMA) with samples from 70 WT (blastemal without anaplasia foci, epithelial and stromal WT type) and 13 normal kidneys. All patients (Series 1 and 2) were treated by neoadjuvant chemotherapy before nephrectomy according to the SIOP-93-01; SIOP 2001 protocol. Patients with localized disease were treated with vincristine and actinomycin D during 4 weeks and those with metastatic disease received vincristine, actinomycin D and doxorubicin during 6 weeks.

All samples were obtained from the Department of Pathology at the Hospital Universitario Virgen del Rocío (Seville, Spain) and HUVR-IBiS Biobank. Approval of the Ethics Committee of our institution was obtained, and written informed consent was obtained before including samples and data in the HUVR-IBiS Biobank. Two experienced pathologists analysed (E.A. and C.S.), identified and confirmed by H&E that the samples were correctly identified as WT and kidneys, and the predominant component in the tumors. Kidneys consisted of renal tissue from the non-tumoral part of the resected specimen following tumoral nephrectomy. Pathologists selected the farthest region from the tumor. Histological stratification of the samples was performed according to SIOP [72] classification. Patients and tumor characteristics are summarized in Supplementary Table 1.

### mRNA and microRNA expression analysis

The expression of selected genes (Supplementary Table 2) was analysed by qRT-PCR using TPT1 as the endogenous control. RNA was isolated from all 22 frozen samples (11 kidneys and 11 blastemal WT), from series 1, with the mirVana miRNA Isolation Kit (Ambion; Life Technologies, NY, USA). The quantity and quality of the total RNA was determined with a Nanodrop ND-2000 Spectrophotometer (Thermo Scientific). Prior reverse transcription was performed using the TaqMan Reverse Transcription Kit (Applied Biosystems; Life Technologies) in the GeneAmp PCR 9700 system and qRT-PCR amplification with the TaqMan Universal PCR Master Mix (Applied Biosystems). We also used qRT-PCR to measure expression of the miR-Let7 family (miR-let7a/b/c) with RNU44 and RNU48 as endogenous controls. Prior reverse transcription was performed using the TaqMan MicroRNA Reverse Transcription Kit (Applied Biosystems) in the GeneAmp PCR 9700 system and qRT-PCR amplification with the TaqMan Universal PCR Master Mix (Applied Biosystems). All qRT-PCR measurements were obtained in a 7900HT Fast Real Time PCR System with the Expression Suite Software v1.0 (Applied Biosystems). Supplementary Table 2 summarizes the genes/transcripts explored and Taqman probes utilized in this study.

### Tissue microarray (TMA) and immunohistochemistry (IHC)

Formalin-fixed paraffin-embedded (FFPE) tissue sections (5 μm) were stained with hematoxylin and eosin. Representative malignant areas from 70 WT patients and 13 kidneys were carefully selected from the stained sections of each tumor. Two tissue cores of one millimeter of diameter were obtained from each sample to build up the TMA.

Five micron-thick TMA sections were dewaxed, rehydrated, and immersed in 3% H_2_O_2_ aqueous solution for 30 minutes to exhaust endogenous peroxidase. Heat-induced epitope retrieval was performed with 1 mM EDTA (pH 9.0) in a microwave oven. Sections were incubated overnight at 4°C with the primary antibodies anti-HMGA2 (Sigma ref.SAB2701959; overnight at 1:4000 dilution); anti-KLF4 (Santa Cruz Biotechnology ref.Sc-20691; overnight at 1:600 dilution); anti-OCT3/4 (Santa Cruz Biotechnology ref.Sc-9081, overnight at 1:400 dilution); anti-NOTCH1 (Santa Cruz Biotechnology ref.Sc-6014, overnight at 1:100 dilution); anti-SOX9 (Santa Cruz Biotechnology ref.Sc-20095, overnight at 1:250 dilution); Snail2/Slug (Cell Signaling Technology ref.9585S, overnight at 1:300 dilution); anti-ABCB4 (MDR3) (Sigma ref.HPA053288; overnight at 1:20 dilution) and anti-Ki-67 (30-9) (Dako ref.IR626; overnight prediluted). Peroxidase-labelled secondary antibodies and 3,3′-diaminobenzidine were applied according to manufacturer’s protocol (EnVision, Dako). Slides were then counterstained with hematoxylin and mounted.

Immuno-stains were scored as low/high expression according to the stain intensity and proportion of stained cells. Sections where the primary antibody was omitted were used as negative controls. Only tumoral cells evaluated as clearly stained were considered to be positive. The IHC results were evaluated by two pathologists (E.A. and C.S.) who scored the average expression of markers in duplicate samples.

### Statistical analysis

Differential expression between kidney and WT were evaluated with the Mann–Whitney *U* test for two groups, and with the Kruskal–Wallis test for more than two groups, followed by Dunn’s multiple comparison post-test. The Spearman’s Rank test was used to quantify the correlation of expression between different genes and markers. Fisher’s and Chi-Square’s exact test were used to evaluate for categorical data. The disease-free survival time was analysed using the Kaplan–Meier estimator, and the differences were evaluated using the log-rank test. Biplots associated with principal component analysis was used to determine the relationship between different variables and Multivariate Spearman’s test was used to determine the correlations between different variables.

For all analyses, data represent mean ± SD; p*-*values of ≤0.05 were considered statistically significant. Univariate analyses were performed using the Prism 4.0 software (GraphPad) and multivariate analyses were performed using the JMP 10 statistical software (SAS Institute, Inc., Cary, NC, USA).

## SUPPLEMENTARY MATERIALS FIGURES AND TABLES


