# Supplementary material for: HMGA2 overexpression predicts relapse susceptibility of blastemal Wilms tumor patients
Source: Oncotarget. 2017 Dec 14;8(70):115290–303. doi: 10.18632/oncotarget.23256 (PMC5777772; doi:10.18632/oncotarget.23256)
Supplement: Supplementary file 1 [file oncotarget-08-115290-s001.pdf]

HMGA2 overexpression predicts relapse susceptibility of blastemal Wilms tumor patients

SUPPLEMENTARY MATERIALS

A

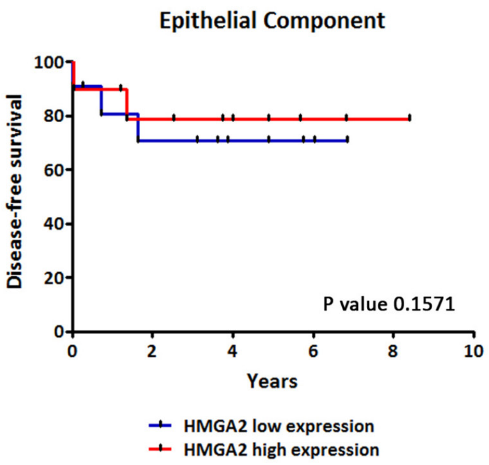

B

	HMGA2
**High expression in epithelial samples**	
Ki67 <10%	7 (58.33%)
Ki67 >10%	5 (41.67%)
**Low expression in epithelial samples**	
Ki67 <10%	10 (90.91%)
Ki67 >10%	1 (9.09%)
**Fisher´s exact test:**	**P value 0.1550**

Supplementary Figure 1: Study of HMGA2 expression in epithelial WT component. **(A)** Kaplan–Meier disease-free survival according to *HMGA2* transcript tumor expression did not show statistically significant differences. **(B)** Relationship between high and low expression of HMGA2 and Ki-67 in epithelial WT component.

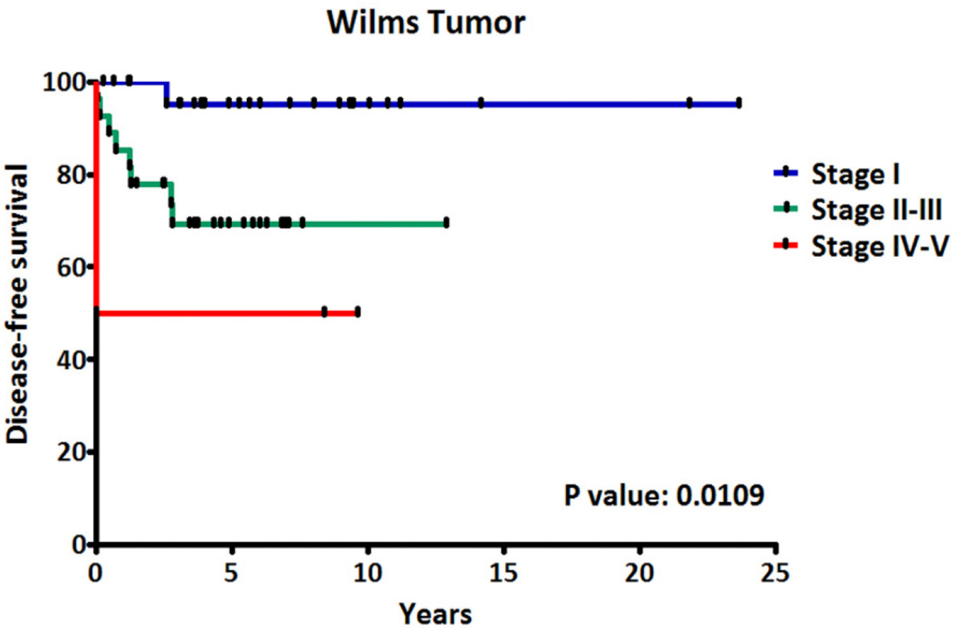

Supplementary Figure 2: Disease-free survival rate according to tumor stage. (A) Tumor stage IV-V showed reduced disease-free survival compared with stage I-II-III.

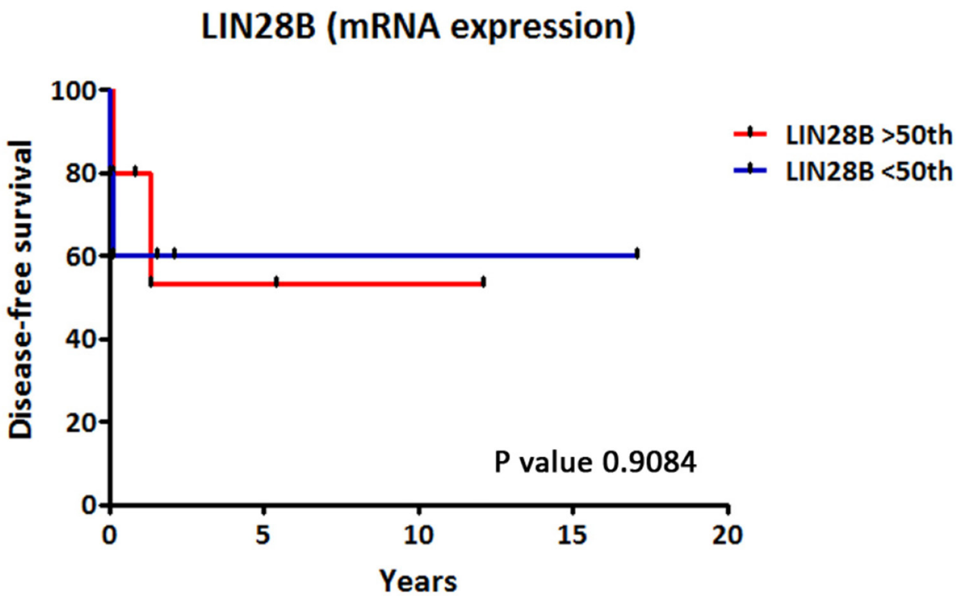

Supplementary Figure 3: Study of *LIN28B* expression in blastemal WT component. *LIN28B* Kaplan–Meier plot with disease-free survival according to the transcript blastemal tumor expression did not show statistically significant differences between low and high expression.

Supplementary Table 1: Clinicopathological features of the tumor samples

	Series 1	Series 2
**Samples**	Frozen Wilms Tumor	Paraffin Wilms Tumor
**Number of samples**	11	70
**Age in years (range)**	2 months- 6 years	5 months- 41 years
**Gender**		
Male	7	34
Female	4	36
**Relapse**		
Present	4	15
Absent	7	55
**Tumor Component**		
Blastemal	11	25
Epithelial	-	36
Stromal	-	9
**Stage**		
I	5	29
II	1	13
III	3	23
IV	2	4
V	0	1

Supplementary Table 2: TaqMan Gene Expression probes and Taqman miRNAs probes for miR-let7 family members -a/ -b/ -c employed for qRT-PCR

GENES	Reference	Amplicon length	PCR conditions
TPT1	Hs02621289_g1	131 bp	* **Rev. transcript.:** 25ºC 5min, 37ºC 120min, 85ºC 5min.
HMGA2	Hs 00171569_m1	65 bp	
LIN28A	Hs00702808_s1	143 bp	* **qPCR:** 95ºC 10min, 40 cycles of 95ºC 30sec and 60ºC 1min.
LIN28B	Hs01013729_m1	130 bp	
MDR3	Hs00240956_m1	73 bp	

miRNAs	Reference		PCR conditions
**miR-let7a**	000377		* **Rev. transcript.:** 16ºC 30min, 42ºC 30min, 85ºC 5min.
**miR- let7b**	000378		
**miR- let7c**	000379		* **qPCR:** 95ºC 10min, 40 cycles of 95ºC 15sec and 60ºC 1min.
**RNU44**	001094		
**RNU48**	001006		
